# A generalizable pre-clinical research approach for orphan disease therapy

**DOI:** 10.1186/1750-1172-7-39

**Published:** 2012-06-15

**Authors:** Chandree L Beaulieu, Mark E Samuels, Sean Ekins, Christopher R McMaster, Aled M Edwards, Adrian R Krainer, Geoffrey G Hicks, Brendan J Frey, Kym M Boycott, Alex E MacKenzie

**Affiliations:** 1 Children’s Hospital of Eastern Ontario Research Institute, University of Ottawa, Ottawa, ON, Canada; 2 Centre de Recherche du CHU-Ste-Justine and Department of Medicine, University of Montreal, Montreal, Canada; 3 Collaborations in Chemistry, Fuquay Varina, NC, USA; 4 Atlantic Research Centre, Dalhousie University, Halifax, NS, Canada; 5 Structural Genomics Consortium, Banting and Best Department of Medical Research, University of Toronto, Toronto, ON, Canada; 6 Cold Spring Harbor Laboratory, Cold Spring Harbor, NY, USA; 7 Manitoba Institute of Cell Biology, University of Manitoba, Manitoba, MB, Canada; 8 Children’s Hospital of Eastern Ontario Research Institute, 401 Smyth Road, Ottawa, ON, K1H 8 L1, Canada

**Keywords:** Orphan disease therapy, Preclinical drug development, Generalizable screening methods, Translational toolbox

## Abstract

With the advent of next-generation DNA sequencing, the pace of inherited orphan disease gene identification has increased dramatically, a situation that will continue for at least the next several years. At present, the numbers of such identified disease genes significantly outstrips the number of laboratories available to investigate a given disorder, an asymmetry that will only increase over time. The hope for any genetic disorder is, where possible and in addition to accurate diagnostic test formulation, the development of therapeutic approaches. To this end, we propose here the development of a strategic toolbox and preclinical research pathway for inherited orphan disease. Taking much of what has been learned from rare genetic disease research over the past two decades, we propose generalizable methods utilizing transcriptomic, system-wide chemical biology datasets combined with chemical informatics and, where possible, repurposing of FDA approved drugs for pre-clinical orphan disease therapies. It is hoped that this approach may be of utility for the broader orphan disease research community and provide funding organizations and patient advocacy groups with suggestions for the optimal path forward. In addition to enabling academic pre-clinical research, strategies such as this may also aid in seeding startup companies, as well as further engaging the pharmaceutical industry in the treatment of rare genetic disease.

## **Introduction**

Single gene disorders, which typically result from mutations having severe effects on gene function, are of particular importance in pediatrics. A significant fraction of pediatric hospital admissions involve genetic conditions [1,2]. The curated Online Inheritance in Man (OMIM) human genetics database currently lists over 3300 genes for which DNA sequence variants have been associated with human disease [3]. The Human Gene Mutation Database (HGMD), using slightly different criteria, lists approximately 3200 such genes [4]. Apart from a fraction of curated variants arising from genome-wide association studies with uncertain functional significance, the majority of these annotations reflect medical conditions with either congenital or childhood onset. Optimal management of patients suffering from these disorders entails both rapid and accurate molecular diagnosis, and, where possible, treatment. The advent of new high throughput, low cost DNA sequencing technologies (so-called next generation systems) has already and will continue to increase the efficiency of new causal gene identification that will facilitate their molecular diagnoses [5]. However, given the tremendous variety of such disorders, their treatment remains an intractable and difficult-to-generalize problem.

**Figure 1:**
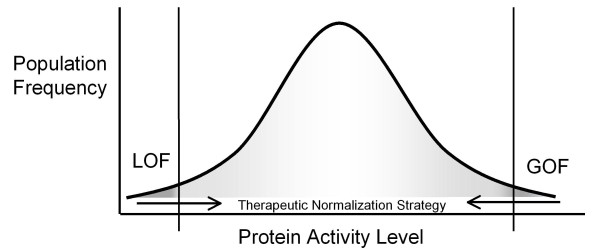
**Normalizing the pathogenic imbalance.** Protein levels and/or activity outside of the physiological normal range usually underlie a monogenic disease. Therapeutic approaches may involve normalizing this imbalance by enhancing the mRNA, protein, or protein activity in disorders caused by LOF mutations and moderating the mRNA, protein, or protein function excess observed in GOF mutations.

Genetic disorders of high penetrance are typically caused by mutations that result in i) loss-of-function (LOF), i.e., a reduction in the level and/or activity of a given protein, usually seen in recessively inherited disorders or ii) gain-of-function (GOF), i.e., an increase in protein level and/or activity with the introduction of a novel pathological function often associated with activation of a pathway, usually seen in dominantly inherited disorders [4]. In broad terms, therapy in either case can be directed at normalizing the pathogenic imbalance; that is to enhance mRNA, protein or protein activity in disorders caused by LOF mutations and moderate the mRNA, protein, protein function, or pathway activity excess observed in GOF mutations (Figure 1). Disorders with a molecular test available for early diagnosis and a presymptomatic window or likelihood of clinical reversibility are potential candidates for therapeutic intervention by this strategy.

In the case of LOF mutations, a functional agonist may be considered, for example via DNA (e.g., gene therapy) or protein replacement (e.g. enzyme replacement therapy). For partial loss of enzymatic function, exogenously increasing levels of the biochemical substrate may be feasible. For some LOF mutations, it may also be possible, with antisense oligonucleotides (ASO) or small molecules, to increase the rate of transcription, correct RNA mis-splicing, allow read through of a premature stop codon, stabilize the transcript, or stabilize or enhance the activity of the mutated protein itself. Finally, for this class of mutation, there may be the option of activating a wild-type homologous gene, thereby recapitulating the function lost in the primary gene mutation, an approach which we term activating the “rescuing paralog”. Conversely, in the case of GOF mutations, a functional antagonist may have clinical value. The therapeutic reduction of the supraphysiologic levels of pathogenic RNA, protein and/or function may be achievable directly by transcriptional or translational inhibition, transcript or protein destabilization, or by direct inhibition of the activity of the protein itself. Alternatively, instead of targeting the gene and its products, the modulation of pathogenically dysregulated pathways by substrate-reduction therapy or product replacement therapy are possible treatments for many LOF and GOF orphan disorders.

### **Therapeutic approaches for loss-of-function (LOF) mutations**

#### ***LOF: Pharmacologic suppression of premature termination codons (PTC)***

The nature of the specific LOF mutation may suggest specific therapeutic approaches, for example a number of drugs are currently being investigated which result in read-through of premature termination codons (PTCs) [6]. These agents reduce ribosome termination at the PTC, resulting in the insertion of a random amino acid and the translation of the remainder of the correct full-length protein. There are a number of limitations to this approach including reduction of the available transcript because of nonsense-mediated mRNA decay, efficient translation and the activity of the protein containing the random amino acid [6,7]. Nonetheless, read through inducing agents show promise for Duchenne muscular dystrophy (DMD) and cystic fibrosis (CF) [8-10] with the potential of eventually treating a wider range of genetic disorders. The current exemplar of nonsense codon suppression is the small molecule PTC124. PTC124 suppresses nonsense mutations at nanomolar concentrations in mammalian cells (but does not appear to alter global protein or mRNA profiles) and is associated with some phenotypic improvement in mouse models with Duchenne muscular dystrophy and cystic fibrosis [6,10,11]. Both diseases have had PTC124 Phase I trials conducted; Phase II clinical trials of PTC124 administered to cystic fibrosis patients that carry nonsense mutations have also been completed, with restoration of measurable CFTR function in half of treated patients and no appreciable impact on CFTR in a second study [12,13]. More recently, PTC124 treatment improved total chloride transport and showed trends toward improvements in pulmonary function and CF-related coughing in a subset of patients with classic CF phenotype and at least one CFTR nonsense mutation allele [14].

In principle, any nonsense mutation that does not trigger significant nonsense-mediated mRNA decay is a candidate for this approach [15]. A challenge in this field is whether there will be a general *in silico* approach to identify small molecules allowing read-through for a given PTC sequence, or whether painstaking and expensive library screening will be needed to identify an agent for each particular disease-causing PTC. Drugs, such as PTC124, that permit read-through of a wide spectrum of stop codons clearly carry a risk of off-target side effects. The expectation in the field is that there may exist unifying principles for the targeting, combined with minimal chemical modification and appropriate dosing, to impart sufficient specificity to minimize such side effects.

#### ***LOF: Therapeutic oligonucleotide based modulation of splicing***

Another class of therapeutic targets comprises the large number of disease-causing mutations occurring in splice site motifs [16]. Such mutations may be intronic or exonic and typically result either in exon skipping and/or use of nearby alternative splice donor or acceptor sites. This results in the deletion of potentially important parts of the encoded protein or, more frequently, exon skipping or mis-splicing resulting in frame shifting errors and resulting aberrant polypeptide with partial or total loss of protein function. Chemically modified oligonucleotides can sterically mask pathogenic splice signals activated by mutation resulting in close to normal levels of mRNA encoding functional protein [17]. Alternatively, in some cases they can be designed to mediate forced splicing exclusion of functionally dispensable in-frame exons containing a pathogenic mutation, resulting in the production of near physiologic levels of internally deleted but partially functional proteins [18]. The application of oligonucleotide-based methods to redirect and modulate pre-mRNA splicing was first demonstrated for β-thalassaemia and now shows promise for both spinal muscular atrophy (SMA) [19] and DMD [20,21]. The design of effective oligonucleotides for this process is undergoing continuous improvement; there now exists both a considerable published body of experience as well as a ‘splicing code’ that relates genomic features to splicing levels in diverse normal and disease tissues, including predictive analyses of how genomic mutations impact splicing [22,23]. There has been recent progress in utilizing the splicing code to identify disease associated mutations in HGM (unpublished data, Frey); it is hoped that the *in silico* identification of disease-causing splice mutations is a realistic goal for the future. Given the increased predictability of the oligonucleotide action on splicing mutations, a central issue is to determine to what extent the absorption, distribution, metabolism, and excretion- toxicity (ADME-tox) characteristics of a given oligonucleotide shall also be predictable; is oligonucleotide ADME-tox a general drug class effect for a given type of chemical modification, minimizing expensive preclinical assessment or is it sequence-dependent, varying from molecule to molecule [24]? Further understanding of this cause and effect relationship will emerge as this approach is applied to more disorders.

#### ***LOF: Pharmacologic modulation of gene activity, mRNA stability and protein function***

Therapeutic upregulation in LOF mutations may be feasible when i) the mutated protein retains some degree of residual function; ii) the disease is caused by a haploinsufficiency; iii) there exists a paralogous normal protein that functionally recapitulates the mutated protein; or iv) there is a protein that mediates a bypass salvage pathway. The general goal in such cases is to increase effective gene function, either directly by modulating the residual expression level or enzymatic activity of the mutated protein itself, or indirectly by upregulating other genes in the genome which themselves may be paralogs of the mutated gene or else active in related biochemical pathways. The introduction of a drug into the complex intracellular and extracellular topography of a human will have, in addition to the impact on its known target, off-target and, from the perspective of orphan genetic disease, potentially beneficial effects. These effects may include the up or down regulation of multiple non-targeted genes, mRNAs or proteins. Indeed, it has been recognized in recent years that small-molecular weight molecules can affect a substantial proportion of the metazoan transcriptome [25,26]. Although such off-target gene modulating effects are currently impossible to predict, the advent of systems biology has begun to permit these effects to be identified with greater effectiveness.

**Figure 2:**
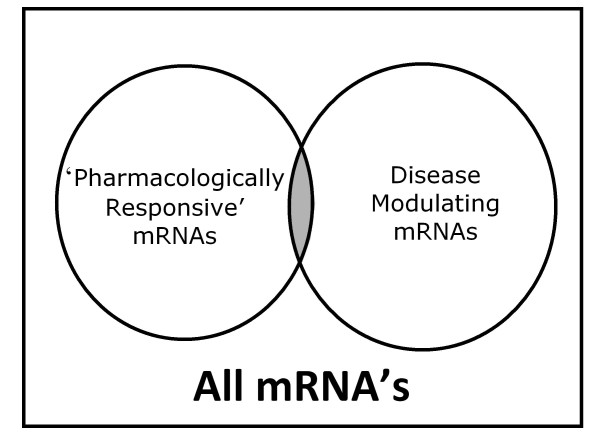
**Pharmacologically responsive therapeutic targets.** mRNA (and thus proteins) which are both pharmacologically responsive and disease modulating represent potential therapeutic targets.

An excellent opportunity to identify beneficial off-target effects comes with novel microarray based databases containing system-wide transcriptome profiles from cell lines grown in the presence of clinically used compounds. This approach has been utilized to catalogue system-wide gene expression profiles elicited by different drugs and drug classes [26-29]. In addition to identifying genomic signatures composed of many transcripts to elucidate drug effects, with these databases one can also ascertain the impact of hundreds of FDA approved drugs on the level of almost every individual transcript in the transcriptome. It is the modulation of individual transcripts that is of particular value for monogenic disorders. For example, we have mined the Johnson and Johnson and Connectivity Map databases [26] for agents that modulate SMN2 mRNA levels, the rescuing paralog for spinal muscular atrophy (SMA; OMIM [253300]), rapidly identifying a role for p38 kinase in the modulation of SMN levels [30]. This approach not only identifies agents that impact transcriptional activity but also those that impact transcript stability as well, such as is seen with p38 activation on SMN2 [30]. The same *in silico* screening approaches can be used to identify agents that upregulate mRNA encoding mutated proteins with residual function, such as seen in milder variants of a recessive disease; given the low levels of protein frequently observed in these disorders, even a modest increase in activity might be anticipated to have a clinically appreciable effect. Similarly, for disorders caused by haploinsufficiency, upregulation of the remaining normal allele may also yield a clinically beneficial effect. It is likely that industry has additional system-wide transcriptional databases that catalogue the impact of large numbers of preclinical and/or clinically used agents. Similarly there exist hundreds of genes which if significantly modulated would have a good likelihood of impacting rare genetic disease. Thus, one goal is to enable improved access to both public and private datasets to expand the set of genes that are both pharmacologically responsive and clinically relevant (Figure 2).

An alternative method of identifying compounds to modify clinically relevant genes is to perform a computational screen for binding sites of pharmacologically inducible transcription factors upstream of the gene of interest. Returning to the example of spinal muscular atrophy, the complementing SMN2 paralog contains in its 5’ region a number of STAT5 kinase binding sites; it has been recently shown that the STAT5 activating hormone prolactin both upregulates SMN protein derived from SMN2 and confers significant survival benefit in a mouse model of SMA [31]. The increasing annotation of system-wide transcription factor binding sites via large CHIP-Seq datasets, wedded with knowledge of agents activating these transcription factors augur well for the wider applicability of this approach.

Limitations do exist to the pharmacologic upregulation of gene activity and mRNA level. The potential strength of systems-wide transcriptional profiling comes with the large number of addresses that are sampled; however, the reproducibility of data for a single microarray address is famously unreliable and it is a case of caveat emptor when utilizing such data. Clearly even before any demonstration of clinical utility, the more independent verification of a given observation from different datasets, the more likely it is to be reliable. Protein modulation is the goal in most rare disease therapeutic approaches and there are not yet systems-wide databases documenting protein levels/activity. Regardless, mRNA serves as a valid proxy for protein level more often than not [32]. In addition, even if a protein:RNA correlation is observed *in vitro*, a given transcript response detected in cell culture may not hold true for a whole organism. It also has to be hoped, when upregulating mutated mRNA and protein, that both are stable and not degraded as can be seen in inherited disorders. Moreover, this approach will not work if the mutated protein has any dominant negative effect. Finally those small molecules which significantly perturb a large proportion of the transcriptome may have a greater incidence of side effects or toxicity than do molecules with milder perturbing effects. For example, histone deacetylase inhibitors such as valproic acid have a significant impact on a substantial proportion of the transcriptome and have simultaneously been suggested for use with a large number of diverse disorders, yet have well documented toxic side effects. The goal of identifying an agent which modulates a specific target in an entirely safe fashion continues to represent a significant challenge.

#### ***LOF: In silico cheminformatic and bioinformatic methods for target proteins***

The complex topology of proteins, and the lack of a comprehensive algorithmic mapping of primary amino acid sequences to three-dimensional structures, makes generalizable approaches to protein functional modulation problematic. Nevertheless, proteins represent a far more frequent therapeutic target for orphan diseases than mRNA. Diverse *in silico* cheminformatic and bioinformatic methods, including target/ligand-based strategies and systems biology methods, are a means of screening large datasets in the hopes of predicting the binding of molecules to proteins [33-40]. Data integration platforms for systems biology, using both ligand and binding site similarity [36,41,42] can help chaperonin identification, possibly involving drug repositioning. In addition, 2D ligand and 3D protein based approaches employing algorithms and networks, have been used to link molecular structure and biological activity [43-45]. The continual development of machine learning methods and databases for drug repurposing also have promise [46-50]. By connecting data on drugs, proteins and diseases, these various computational methods may enable repurposing of molecules and possibly enable *in vitro* screening efforts for orphan disease therapy [51-55]. Implicit in using these molecular, protein and gene expression databases is the requirement that the data are sufficiently free from error so as not to hamper the quality of the prediction [56].

Perhaps the best categorized area of protein modulation is pharmacologic chaperone therapy: small molecules that specifically bind to target proteins modulating folding and/or stability [57-61]. This approach is currently either being investigated or used for various orphan conditions, particularly lysosomal storage disorders [60,62]. Pharmacological chaperones stabilize the folding of mutant proteins and allow for correct trafficking of the enzyme, often resulting in increased egress from the Golgi to the cytosol or cell surface. Molecules that would be predicted to inhibit the lysosomal enzyme counterintuitively often serve as protein stabilizers and de facto enzyme activators with therapeutic potential; presumably the negative effect of competitive inhibition for substrate binding is more than offset by the positive effect of increased correct protein folding on total enzyme produced [61]. Such observations serve as a key starting point for drug development for other enzymes besides those involved in lysosomal storage diseases; a considerable proportion of molecularly characterized monogenic genetic disorders involve enzymes and present with aberrant, often life-threatening metabolomic profiles (e.g. phenylketonuria).

High content cellular screening for compounds leading to correction of mutated trafficking anomalies, may be one robust route to active pharmacologic chaperones although given the cost of these approaches the preferable scenario would include a relatively common or recurrent causal mutation (which are often seen in local and even large founder populations) and a good *in silico* lead into a compound class.

In this context, RNAi approaches with cellular screens for protein localization may identify currently unappreciated genes that would have a broad impact on folding of mutated polypeptides, irrespective of specific mutation or even particular protein involved. Once identified, altered regulation of such genes by the approaches described above might be clinically beneficial for a wide range of genetic disorders involving missense mutations and aberrant protein folding. Finally, modulation of the specific function of a mutated protein can be obtained, as has recently been documented in a successful clinical trial of CFTR conductance modulators for cystic fibrosis [63].

#### ***LOF and GOF: Pathogenic pathway modulation***

Regardless of the underlying genetic lesion, there is usually a dysregulation of a biochemical/metabolic/intracellular pathway that is the primary pathogenic driver of a given disorder. Identifying the pathway underlying disease pathogenesis, along with an understanding of the nature of the dysregulation, brings with it the opportunity for therapeutic pathway modulation. The intervention is clearly contingent upon the specific pathway involved and thus is less open to generalizable methods, although the approach may allow for a common intervention to be used for different disease genes that are within the same pathway. Nonetheless, dysregulated pathways may for many disorders, represent the most accessible and relevant therapeutic targets. The pathogenic effect may be as simple as a build-up of a toxic substrate or dearth of a critical product in a metabolic pathway; in such cases, means of reducing the former and increasing the latter are obvious therapeutic approaches. For example, the zinc deficiency observed in acrodermatitis enteropathica can be addressed by dietary supplementation [64,65], while the accumulation of a toxic metabolite may be addressed by dietary means, such as the phenylalanine restriction used to treat phenylketonuria [62]. Where dietary modification is either not possible or insufficient, modulation of the activity of an alternate pathway may be employed as a possible adjunctive therapy. One example is the recessive Tay Sachs disease (TSD), resulting from mutation in the *HEXA* gene encoding the α subunit of the lysosomal enzyme, β-hexosaminidase [66]. The enzyme mediates breakdown of GM2 ganglioside; its loss results in GM2 accumulation and progressive neurodegenerative disease. There exists a metabolic bypass, mediated by Neu1/PPCA or Neu3 sialidase enzymes, which can also break down GM2 ganglioside; thus, the pharmacologic induction of these genes might be therapeutic in TSD [67].

#### ***LOF: Other translational approaches***

Completing the list of promising orphan disease treatments for LOF mutations are enzyme replacement and gene replacement therapies. Enzyme replacement is a proven approach (although neurologic correction remains elusive due to the impermeant blood brain barrier) and gene therapy after a period of disappointments appears to be coming of age. Unfortunately these approaches remain research intensive and likely prohibitively expensive for the treatment of a large number of very rare disorders. Extensive research is ongoing within these fields and the hope is that they will eventually become more viable options, but they are not the focus of our current translational path.

### **Therapeutic approaches for gain-of-function (GOF) mutations**

XGain-of-function mutations, usually dominantly inherited, are caused by a supra- and thereby pathophysiologic level of mRNA/protein and/or a novel pathologic gain-of-function. There are a number of methods to moderate the mRNA, protein, novel protein function, or pathway activity excess observed in GOF mutations. Oligonucleotides generated using novel second- and third-generation backbone-modifications can reduce RNA and thus protein by at least 11 distinct mechanisms, including the direct sequence-specific steric block by hybridization to pre-mRNA (RNAi), the alteration of pre-mRNA splicing, hybridization to processed mRNAs thereby preventing ribosome recruitment, and the blockage of protein translation [68]. Oligonucleotides can also inhibit the actions of non-coding RNAs such as microRNAs. In addition to antisense oligonucleotides (ASOs), one can also screen for pharmacologic (in this case down regulatory) modulation of the transcriptome such as outlined above for LOF mutations or screen for pharmacologic direct inhibitors of protein function also using the *in silico* approaches outlined in the previous section. Screens for small molecules binding to the GOF protein itself could be done both through high-throughput screens or *in silico* modeling.

If there is a pathogenic activation of a given pathway, then other means of inhibiting that particular cascade can be attempted. In general terms, this concept was described above in “pathogenic pathway modulation”. For GOF disorders, the dominantly inherited kinasopathies represent an instructive disease class in this regard; the majority of these involve a gain of pathogenic function frequently leading to a constitutive activation of a signaling pathway. This has resulted in a rare disease drug repurposing opportunity, as some of these pathways have also been implicated in oncogenesis and are targets for kinase inhibitor programs at pharmaceutical companies. One example is the autosomal dominantly inherited human developmental syndromes termed the RASopathies caused by (usually) activating mutations of the RAS-RAF-MEK-ERK MAPK pathway [69,70]. The most common of these is Noonan syndrome, characterized by proportional short stature, facial dysmorphia, and cardiovascular abnormalities. A clinical trial with the MEK1/2 inhibitor (also being trialed for several malignancies) for treatment of cardiomyopathy in adults with Noonan syndrome has recently been launched (ClinicalTrials.gov Identifier: NCT01556568); the hope is that other kinase inhibitors will find their way from cancer trials into orphan disease trials.

**Figure 3:**
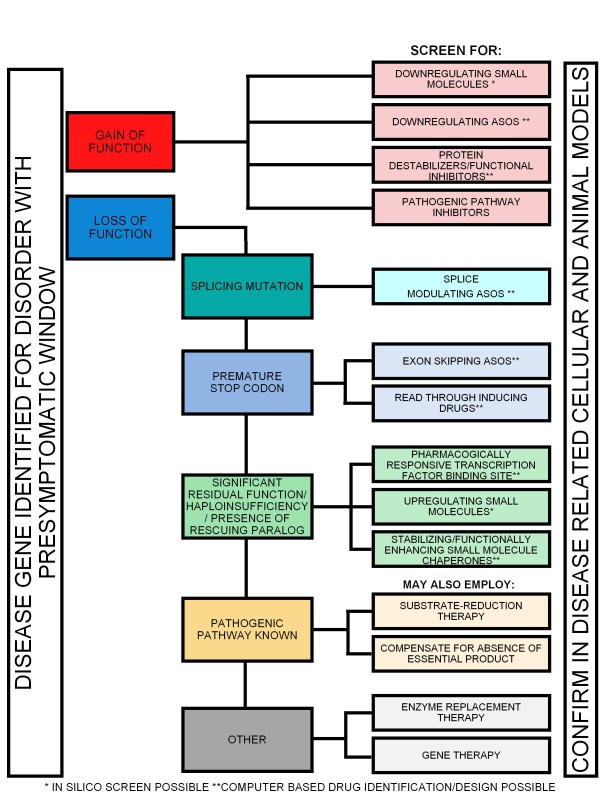
**Orphan disease translational pathway.** Schematic of possible orphan disease therapeutic avenues depending on nature of mutation, disease mechanism, and information mined from existing datasets. *In silico screen possible. **Computer based drug identification/design possible.

**Figure 4:**
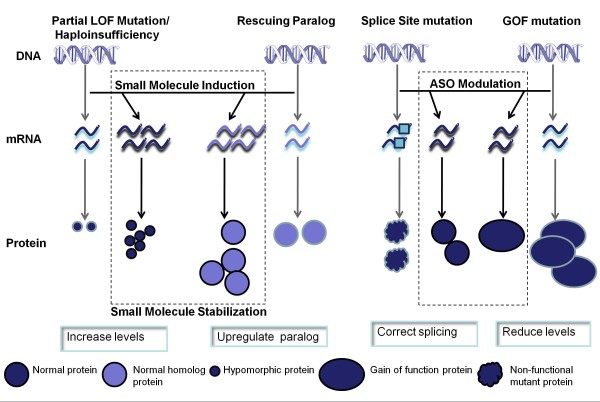
**Therapeutic approaches based on small molecules and ASOs.** Small molecules have the potential to regulate the expression of genes. Haploinsufficient or partially functioning proteins can be upregulated to compensate for the reduced activity. If a non-functional mutated gene has a homolog with overlapping function, the homolog can be upregulated to partly compensate for the non-functioning protein (termed rescuing paralog). ASOs have the potential to modify pre-mRNA splicing or expression. Cryptic splice sites caused by mutations can be blocked to correct splicing. Conversely, genes with overexpression or with gain-of-function mutations can be downregulated.

A schematic outlining the approaches and the proposed translational research path for both LOF and GOF classes of mutation is summarized in Figures 3 and 4.

### **The path following potential therapeutic agent identification**

Following the *in silico* identification of a putative therapeutic agent, the confirmation of the proposed effect both in cell cultures and *in vivo* will be required. We have found that this assessment can be done both in wild-type and patient cell lines (using either immortalized fibroblasts or lymphocytes, or with new induced pluripotent cell (iPS) technology) as well as wild-type animals in addition to animal models of a given disorder. For example, an increase of the protein level and/or activity in LOF mutations (or diminution of these parameters for GOF mutations) in wild-type cells and animals can be suggestive of a true effect. The presence of a scorable phenotype in patient cell cultures as well as a biomarker in the animal models (and naturally the human disease), help further in the assessment of the proposed therapy. The summary outlined in Table 1 highlights the various resources that will be of assistance for this phase of disease pathogenesis and preclinical research.

**Table 1:** Translational toolbox: Conditions and resources that will enable effective orphan disease translational research


**Critical Information**	Known Gene
	Known Inheritance (dominant or recessive)
	Known Mechanism (LOF or GOF)
**Helpful Information**	Existence of a presymptomatic window or likeliness of clinical reversibility
	Dysregulated pathway known
	Diagnostic assay associated with primary defect available
	Purified protein available
	Protein crystal structure known
	Antibody directed against protein available
	Previous screen related to disease gene exists (screen for pharmacologic modulation, ASO screening, etc)
	Gene under control of transcription factors responsive to drugs
	Scorable cell culture phenotype exists
	Animal or other model available
	Scorable biomarker reflecting disease state exists (metabolomic, transcriptomic marker, etc)
	Disease management protocol in use

## **Conclusion**

Translational research for rare diseases is clearly a resource intensive undertaking, both in terms of time and real cost. Therefore, as much as possible, we have attempted to define an approach that relies on databases and computational analyses prior to the more expensive experimental validation of potential therapies (examples of potential new therapies for rare diseases identified using these approaches are listed in Table 2). This is especially important when a disease is so rare as to render the prospects of any commercial profit from a therapy, regardless of its effectiveness, unlikely. Thus, an important issue is the question of generalization of approach and minimization of costs. Key steps in this direction include access to system-wide datasets, compounds and reagents for the orphan disorder research community, advances in both systems biology and computational prediction of small molecule-macromolecule interaction, the identification of additional generalizable therapeutic approaches and ultimately more collaboration.

**Table 2:** Examples of computational technologies used for rare disease drug discovery

**Strategy**	**Rare disease**	**Computational Technology**	**Drug**	**Reference**
Small Molecule Upregulation	Spinal Muscular Atrophy	Connectivity Map	Anisomycin	[30]
Small Molecule Upregulation	Spinal Muscular Atrophy	Transcription factor binding site identification	Prolactin	[31]
Chaperone: drug safety predictions	Gaucher disease	Leadscope	Core structures of aminoquinoline, sulfonamide, and triazine	[71]
Chaperone: identify binding sites and compounds	Huntington disease	AutoDock, Patch Dock Server, CastP	Metoprolol, minocyclines, and 18 F fluorodeoxyglucose	[72]
Drug similarity predictions	Neurodegenerative disorders due to protein misfolding	Mode of Action by Network Analysis, MANTRA	Fasudil	[27]
Prediction of which mutations respond to treatment	Fabry disease	Position specific substitution matrix	1-deoxy-galactonojirimycin	[73,74]

### **Accessibility to a greater pharmacophoric compound library space, data currently existent in pharmaceutical companies and readily available reagents**

There currently exists, in major pharmaceutical companies, extensive databases describing the system-wide transcriptional response to thousands of compounds, the vast majority of which have not seen clinical use. Although most of these compounds are designed for proteases, kinases and G-protein coupled receptors, mining these datasets and subsequent access to those compounds that modulate orphan disease related transcripts might serve as a source of rare disorder therapeutic leads. In addition there does not yet exist a definitive gold standard transcriptomal database to mine computationally for FDA approved drugs; the configuration of such an entity would be of significant value. Finally, but of no less importance, is the key set of reagents that needs to be generated for as many orphan diseases as possible to encourage research in that area by the community. Purified protein, crystal structures, antibodies as well as cellular and animal models, will both help advance the prospects of meaningful translational research as well as enlist new research teams into the field of rare genetic diseases.

### **Computational prediction of small molecule-macromolecule interaction**

Computational advances are also needed in the prediction of small molecule-RNA/protein interactions including the identification of agents that suppress PTCs, bind and then stabilize, destabilize or antagonize protein activity. Improved prediction of the splicing impact of relevant genomic mutations and of optimal antisense oligonucleotides that may reverse the effects of such mutations would also be of value.

### **Additional generalizable therapeutic approaches**

A key aspect of any broadly successful approach for this class of disease will be to conceive and employ generalizable methodologies wherever possible. Some examples of these include the possibility of generalizing ADME-toxicology for oligonucleotides, identifying novel broad spectrum PTC suppressor compound classes or possibly identifying a druggable pathway that allows persistence of higher levels of non-toxic, partially functional misfolded proteins.

While the approaches we propose here are credible and feasible, the prospect of a rapid configuration of numerous effective orphan disease therapies should be viewed in perspective. Two of the most “common” rare diseases, CF and DMD, have been the subject of many years and millions of dollars of pre-clinical and clinical assessment; indeed many of the approaches outlined above have been pioneered in the analysis of these disorders, yet there is today still not an effective therapy in routine clinical use for either disorder. Nonetheless, we hope that among the many genetic disorders that have been or will shortly be molecularly characterized; there will be some that are tractable to the approaches reviewed here. At a minimum, the generation of a standardized toolbox will help to move a larger number of disorders closer to the day of effective therapy.

## **Abbreviations**

ADME-tox, Absorption distribution, metabolism, and excretion – toxicity; ASO, Antisense oligonucleotide; CF, Cystic Fibrosis; DMD, Duchenne muscular dystrophy; GOF, Gain-of-function; LOF, Loss-of-function; PTC, Premature termination codon; SMA, Spinal muscular atrophy; TSD, Tay Sachs disease.

## **Competing interests**

The authors declare that they have no competing interests.

## **Authors’ contributions**

All authors contributed to the literature review and the writing of the article. All authors read and approved the final manuscript.
